# Functional impairment and post-stroke depression: a 6-month longitudinal study

**DOI:** 10.47626/2237-6089-2022-0589

**Published:** 2025-04-07

**Authors:** Larissa P. Borlina Beltrami, Paula Teixeira Marques, Francisco Jaime Lopes Barbosa, Viviane H. Flumignan Zetola, Marcos Christiano Lange, Raffael Massuda

**Affiliations:** 1 Universidade Federal do Paraná Curitiba PR Brazil Programa de Pós-Graduação em Medicina Interna e Ciências da Saúde (PPGMICS), Universidade Federal do Paraná (UFPR), Curitiba, PR, Brazil.; 2 UFPR Hospital de Clínicas do Paraná Complexo Hospital de Clínicas Curitiba PR Brazil Hospital de Clínicas do Paraná, Complexo Hospital de Clínicas, UFPR, Curitiba, PR, Brazil.; 3 UFPR Departamento de Psiquiatria Curitiba PR Brazil Departamento de Psiquiatria, UFPR, Curitiba, PR, Brazil.

**Keywords:** Stroke, post-stroke depression, predictive factor

## Abstract

**Objective:**

In recent decades, there have been considerable advances in treatment and prevention of acute ischemic stroke (IS). However, even after treatment, approximately two-thirds of patients with IS have some degree of disability that requires rehabilitation, along with an increased likelihood of developing psychiatric disorders, particularly depression. The objective of this study was to determine the predictors of post-stroke depression in a 6-month period in patients with IS.

**Methods:**

Ninety-seven patients with IS without previous depression were included in the study. The study protocol was applied during hospitalization and at 30, 90, and 180 days after hospital discharge. A binary logistic regression was then conducted. Age, sex, marital status, occupation, educational level, thrombolysis, National Institute of Health Stroke Scale (NIHSS), modified Rankin Scale (mRS) score, Barthel Index (BI), and Mini-Mental State Examination (MMSE) score were included as independent variables.

**Results:**

Of the 97 patients, 24% of patients developed post-stroke depression. In the longitudinal follow-up, an mRS score of > 0 was the lone significant predictor of development of depression (odds ratio [OR] = 5.38; 95% confidence interval [95%CI] 1.25-23.12; p < 0.05).

**Conclusion:**

Our results showed that in patients without previous depression, functional impairment of any degree is associated with a 5-fold greater chance of development of depression in the first 6 months post-stroke compared to patients without functional impairment.

## Introduction

Stroke involves an interruption of blood flow in a particular area of the central nervous system. Symptoms of stroke appear suddenly, persist for more than 24 h, and are related to the brain territory affected by ischemia due to arterial blood flow obstruction or cerebral hemorrhage due to cerebral blood vessel disruption. The patient may exhibit changes to consciousness level, eye movements, face movements, muscle strength, sensitivity, speech, balance, gait, and attention.

Globally, stroke incidence rates have remained stable and mortality rates have declined over the last 20 years. As a consequence, there has been an increase in the number of stroke survivors with disability-adjusted years of life (DALYs) lost due to stroke.^1^ Stroke is one of the main factors of functional impairment. Six months after stroke, 26% of patients older than 65 years have become dependent on others to perform their activities of daily living, and 46% of such patients have cognitive deficits.^2^

Despite treatment, approximately two-thirds of stroke survivors have some degree of disability that requires rehabilitation,^3^ along with an increased likelihood of developing psychiatric disorders, particularly depression, which can occur in up to one-third of patients at some point of clinical follow-up.^4^ The main clinical manifestations of this "invisible" post-stroke deficiency are mood disorders and cognitive impairment.^5^

Patients with post-stroke depression have an increased risk of worse functional outcomes, recurrent stroke events, worsening quality of life, and increased mortality.^4,6^ Studies have identified previous history of depression, cognitive deficits, earlier episodes of stroke, severe neurological deficits and disability, family history of psychiatric disorder, and female gender as predictors of post-stroke depression.^6-8^ Many studies also include hemorrhagic stroke and a previous history of mood disorders as predictors of post-stroke depression.^7^

Our study aimed to evaluate whether gender, age, functionality at hospital discharge after stroke, treatment with thrombolysis, and Mini-Mental State Examination (MMSE) score are predictors of depression within 6 months following ischemic stroke (IS) in patients without a previous history of mood disorders.

## Methods

### Sample selection

Patients were selected who had a first-time diagnosis of IS and were admitted to the Stroke Unit at the Hospital de Clínicas (HC), Universidade Federal do Paraná (UFPR), Curitiba, Brazil from May 2016 to August 2017. All patients underwent clinical evaluation for IS diagnosis, followed by a computed tomography (CT) scan. Patients were indicated to undergo thrombolysis if they met the clinical criteria based on the onset of stroke symptoms: the patient had been in good medical condition 4.5 h before the onset of symptoms, cranial tomography showed no signs of hemorrhage, absence of dysglycemia or blood pressure peaks above 185/110 mmHg, and absence of contraindications.

The exclusion criteria for our study were: patients with a history of previous IS; those under 18 years of age; those with a previous diagnosis of mental disorders, including mood disorders; and those with aphasia or language limitation and/or impaired understanding of the consent form.

### Ethical considerations

The study was approved by the HC UFPR ethics committee. All participants signed a free and informed consent form that had been approved by the HC UFPR Research Ethics Committee.

### Protocol for clinical and psychiatric evaluation

The protocol was applied at hospital discharge after clinical stabilization of the patient's condition and again at the Cerebrovascular Diseases Outpatient Clinic at 30, 90, and 180 days after hospital discharge.

The National Institute of Health Stroke Scale (NIHSS) comprises 15 items providing a quantitative measure of the main components of the standard neurological examination, including consciousness level, eye movements, visual fields, facial motricity, muscle strength, sensory function, language, coordination, speech, and attention.^9,10^ Performance on each item is scored on an ordinal scale ranging from 0 to 2, 0 to 3, or 0 to 4. Item scores are summed to provide a total score ranging from 0 to 42; the higher the score, the more severe the stroke intensity.^11^

The Rankin scale (RS) is used to evaluate stroke outcomes.^12,13^ It assesses overall disability, in particular physical disability, functional disability, and the need for assistance. The modified RS (mRS) has seven grades, from 0 to 6, where grade 0 indicates no symptoms, grades 1 to 5 indicate a progressive worsening of the patient's incapacity to conduct activities of daily living, thus requiring caregiver attention, and grade 6 indicates death.^10^

The Barthel index (BI) is commonly used to measure stroke victims’ disability or dependence on others for activities of daily living. The items evaluated are categorized into a self-care group (food, personal hygiene, and sphincter control) and a mobility-related group (ambulation, transfers, and climbing stairs).^14^

The Mini-Mental State Examination (MMSE) is used for screening and initial diagnosis of cognitive alterations. It can also be used to analyze disease progression and evaluate treatment responses in patients with dementia syndrome. It is also applied to understand the influence of educational level on total MMSE score. The cutoff scores for normality proposed by Brucki et al.^15^ were 20 for illiterate subjects, 25 for subjects with 1 to 4 years of education, 26 for 5 to 8 years of education, 28 for 9 to 11 years of education, and 29 for individuals with more than 11 years of education.

The Mini International Neuropsychiatric Interview (MINI) is a semi-structured interview for diagnosing psychiatric disorders listed in the Diagnostic and Statistical Manual of Mental Disorders, 4th edition (DSM-IV) and the International Classification of Diseases, 10th revision (ICD-10)^16^ through a short and accurate questionnaire that can be used for multicenter clinical trials and clinical practice in psychiatry.^17^

### Statistical analysis

Statistical analysis was performed using JAMOVI 2.3.19 software. The results were expressed as mean, standard deviation (SD), median, and minimum, and maximum values (quantitative variables) or as frequency and percentage (categorical variables). A binary logistic regression was performed to evaluate the predictors of post-stroke depression. The dependent variable was presence of depression at some point in the 6-month period after IS. The independent variables were age, gender, educational level, thrombolysis, MMSE score, low MMSE score for educational level, NIHSS score at hospital discharge, mRS score, and BI. The requirements for regression analysis of absence of multicollinearity with tolerance greater than 0.1 and variance inflation factor (VIF) less than 10 among the independent variables were met.

## Results

A total of 101 patients were selected. Three patients were excluded because of previous psychiatric history and one patient diagnosed with hemorrhagic stroke was excluded. Finally, 97 patients were included in the study. Table 1 shows the sociodemographic data and clinical characteristics of the patients included in the sample.

**Table 1:** Sociodemographic characteristics of the included patients at baseline

		Patients with IS (n = 97) n (%)
Age (mean ± SD)		59.6 ± 14.6
Sex (M/F)		57 (58.8)/40 (41.2)
Marital status (partner/no partner)		65 (67.0)/30 (31.0)
		
Educational level (years)		
	up to 4	46 (47.4)
	5-11	32 (33.0)
	> 11	19 (19.6)
		
Thrombolysis (yes/no)		36 (37.1)/60 (61.9)
MMSE scores (mean ± SD)		24.2 ± 3.7
MMSE scores for educational level (normal/low)		40 (40.2) 55 (56.7)
NIHSS		1.0 ± 2.0
		
Modified Rankin scale (mRS)		
	0	42 (43.0)
	1	25 (25.8)
	2	19 (19.3)
	3	4 (4.1)
	4	7 (7.2)
	5	0 (0.0)
	6	0 (0.0)
		
BI (mean ± SD)		99.2 ± 2.1

BI = Barthel index; F = female; IS = ischemic stroke; M = male; MMSE = Mini-Mental State Examination; mRS = modified Rankin Scale; NIHSS = National Institutes of Health Stroke Scale; SD = standard deviation.

**Figure 1:**
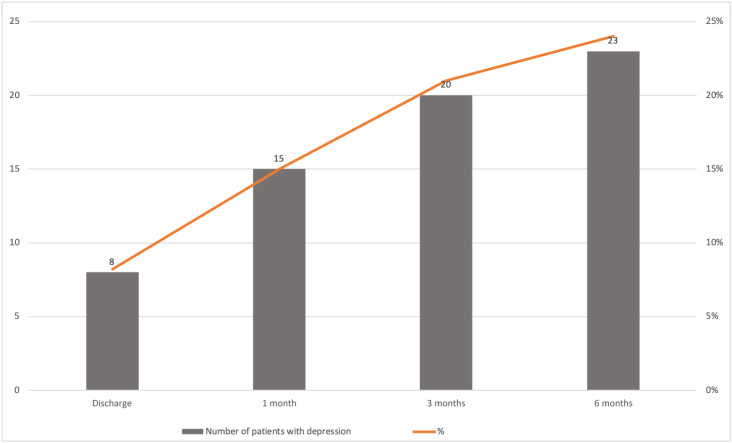
Cumulative incidence of depression after ischemic stroke.

Among the patients included in the study, 23 patients (24%) experienced depression at some point of time in the 6-month period (Figure 1). Thirteen patients (13%) were lost to follow-up, six (6%) of whom were lost at the first follow-up; four (4%) at the second follow-up, and three (3%) at the third follow-up. We adopted the last observation carried forward (LOCF) approach to maintain these patients’ data.

We performed a logistic regression analysis with depression at any time in the 6-month period as a dependent variable. Age, gender, educational level, thrombolysis, NIHSS score, BI, MMSE score, low MMSE score for educational level, marital status, and mRS score were included as independent variables. However, because of the sample size, we adjusted the mRS score for presence or absence of functional impairment (binomial variable), where a score of 0 represents the absence of functional impairment. In contrast, a score of 1 or more represents some functional impairment. The binary logistic regression analysis revealed that only an mRS score greater than 0 was statistically significant as a predictor of development of depression during the longitudinal follow-up of patients after IS (odds ratio [OR] = 5.38; 95% confidence interval [95%CI] 1.25-23.12; p < 0.05). The other independent variables were not statistically significant for predicting development of depression (Table 2). None of the included variables showed multicollinearity.

**Table 2:** Logistic regression analysis with depression as the dependent variable

Variable	Coefficient	Wald	p-value	OR	95%CI for OR
Age	0.003	0.175	0.90	1.00	0.96-1.05
Sex	-0.552	0.881	0.35	0.55	0.18-1.67
Marital status	0.178	0.088	0.77	1.20	0.37-3.87
Educational level (up to 4 years/5-11 years)	1.088	1.190	0.28	2.97	0.42-20.95
Educational level (up to 4 years/> 11 years)	0.256	0.75	0.79	1.29	0.21-23.12
Thrombolysis	-0.21.9	0.138	0.711	0.80	0.25-2.55
mRS (0/> 0)	1.682	5.105	0.02	5.375	1.25-23.12
NIHSS scale	-0.009	0.005	0.95	0.99	0.76-1.29
BI	-0.072	0.354	0.55	0.93	0.73-1.18
MMSE scores	0.120	0.862	0.35	0.93	0.88-1.46
MMSE score for educational level (normal/low)	-0.434	0.285	0.593	0.65	0.13-3.18

95%CI = 95% confidence interval; BI = Barthel index; MMSE = Mini-Mental State Examination; NIHSS = National Institutes of Health Stroke Scale; OR = odds ratio; mRS = Modified Rankin Scale.

## Discussion

According to the results of our study, the cumulative incidence of depression at 6 months in patients with IS was 24%. This finding was similar to the frequency of mood disorders reported in previous literature.^3,4,6^ Studies that evaluated patients after stroke showed that degree of disability, stroke severity, previous history of depression, and cognitive deficits were factors related to the risk of subsequently developing depression.^8,18-20^ The association between depression and stroke severity supports the hypothesis of a direct relationship between neurological damage and mood disorder.^8^

In our study, in patients with functional impairment, an mRS score of > 0 increased the likelihood of developing depression during the first 6 months after IS occurrence by more than 5 times compared to the likelihood in patients without functional impairment. A similar result was reported by Schwab-Malek et al.,^21^ who found that depressive symptoms were present in 16% of patients with mRS scores of 0-2 at 3 months after stroke. A prospective study by Snaphaan et al.^22^ evaluated 420 patients and observed an increased risk of post-stroke depression in patients with an unfavorable functional result identified by an mRS score ≥ 2 (OR = 4.54; 95%CI 2.29-9.01).

The present study did not find associations between other sociodemographic factors or stroke symptoms and development of depression. Previous studies have reported divergent results for the associations between gender and age and development of depression.^18,23^ However, a meta-analysis by Shi et al.^24^ showed that gender and age > 70 years were predictors of development of depression after stroke.

In our study, no significant associations were observed between depression and stroke severity (reflected by the NIHSS score) or between depression and impact on activities of daily living (assessed by the BI). One possible explanation for this finding is that our sample population had low stroke severity and low functional impairment, which is because only patients with IS and lower NIHSS scores were included in our study. When patients with extensive severity of IS and hemorrhagic stroke and those with greater functional impairment are included in the study cohort, then NIHSS score and BI also seem to predict development of depression, in addition to mRS.^25^

Thrombolysis, MMSE scores, and cognitive performance below that expected for educational level were also not associated with development of depression within the 6 months of follow-up. In a meta-analysis published by Perrain et al.,^7^ cognitive impairment was not found to be a risk factor associated with development of depression in longitudinal studies. The causes of the increased frequency of depressive episodes after stroke are currently under investigation. Factors such as functional impairment and increased brain inflammatory processes have been suggested as the causes.^26^

Our study has some limitations, including follow-up in a single hospital and a limited number of patients. The selection of patients without aphasia for diagnostic interviews reduced the stroke severity level of the patients enrolled on the study and probably led to lower rates of depressive episodes. Additionally, we did not include stroke location in the brain as an independent variable in our analysis. The strengths of our study are its longitudinal follow-up of patients, low rates of follow-up losses, and use of structured interviews to diagnose depression.

Post-stroke depression is a common condition and may affect up to one-third of survivors at any time during clinical follow-up. The clinical course of post-stroke depression is dynamic and its pathophysiology is poorly understood. Post-stroke depression is associated with worse functional outcomes, poor treatment, and increased mortality risk. Identification of risk and treatment factors may improve patients’ quality of life and outcomes after stroke.^3,4,20^
